# Structural basis of poxvirus A16/G9 binding for sub-complex formation

**DOI:** 10.1080/22221751.2023.2179351

**Published:** 2023-03-01

**Authors:** Fanli Yang, Sheng Lin, Zimin Chen, Dan Yue, Ming Yang, Bin He, Yu Cao, Haohao Dong, Jian Li, Qi Zhao, Guangwen Lu

**Affiliations:** a West China Hospital Emergency Department (WCHED), State Key Laboratory of Biotherapy, West China Hospital, Sichuan University, Chengdu, People’s Republic of China; b Disaster Medicine Center, West China Hospital, Sichuan University, Chengdu, People’s Republic of China; c Laboratory of Aging Research and Cancer Drug Target, State Key Laboratory of Biotherapy and Cancer Center, National Clinical Research Center for Geriatrics, West China Hospital, Sichuan University, Chengdu, People’s Republic of China; d School of Basic Medical Sciences, Chengdu University, Chengdu, People’s Republic of China; e College of Food and Biological Engineering, Chengdu University, Chengdu, People’s Republic of China

Dear Editor,

On July 23rd, 2022, the sudden monkeypox outbreak was declared as a Public Health Emergency of International Concern by the World Health Organization [1]. The latest epidemiological data has revealed a total of 84,733 laboratory-confirmed monkeypox cases with 80 deaths in 110 countries, areas and territories [1], highlighting an unexpected poxvirus-associated pandemic globally. While the COVID-19 pandemic is still surging, the emergence of monkeypox virus infection has unavoidably posed a greater threat to social economy and public health. Poxviruses and the related diseases once again draw worldwide attention after the eradication of smallpox in the late twentieth century [2]. Entry, within the viral life cycle, is the first step for a virus to set up infection and also the most important target for the development of antiviral therapy. It is therefore an urgent issue to characterize the structural features of key viral proteins involved in poxvirus entry.

Poxviruses are a group of enveloped viruses, the entry of which require the fusion between viral envelop and cell membrane. For most of other enveloped viruses, their attachment and entry just depend on one or a few proteins. In poxviruses, however, its fusion process is mediated by a large proteinaceous machinery, which is designated as the entry-fusion complex (EFC) [3,4]. It is believed that poxvirus EFC is composed of at least eleven virus-encoded protein subunits, among which the A16 and G9 subunits (nomenclatures of A16, G9 and other proteins refer to vaccinia virus, a prototype membrane of *Orthopoxvirus* genus in *Poxviridae* family [5]) form a sub-complex and play a key role in EFC assembly and function. In addition, the A16/G9 sub-complex is also reported to interact with the viral A26 protein and A56/K2 complex, which could in turn moderate the fusion process [6,7]. In recognition of the important functions of the A16/G9 sub-complex, the atomic structure of this sub-complex, however, remains uncharacterized.

In this study, we reported the crystal structure of the A16/G9 sub-complex from the vaccinia virus. As expected, the two proteins and their homologs of known orthopoxviruses individually exhibit high sequence similarities (Supplementary Figure S1). To verify the sub-complex formation, A16-ectodomain engineered with a C-terminal His-tag and G9-ectodomain with a C-terminal Strep-tag were co-expressed in insect cells (Figure 1(A,B)). Expectedly, the two proteins were easily co-purified during Ni-NTA affinity chromatography and remained bound as a stable complex in solution during gel-filtration chromatography (Figure 1(B,C)). The structure of the A16/G9 sub-complex were subsequently solved via X-ray crystallography. The final structure, with a resolution of 2.7-Å, was refined to *R*_work _= 0.243 and *R*_free_ = 0.273, respectively (Supplementary Table S1).

**Figure 1.**
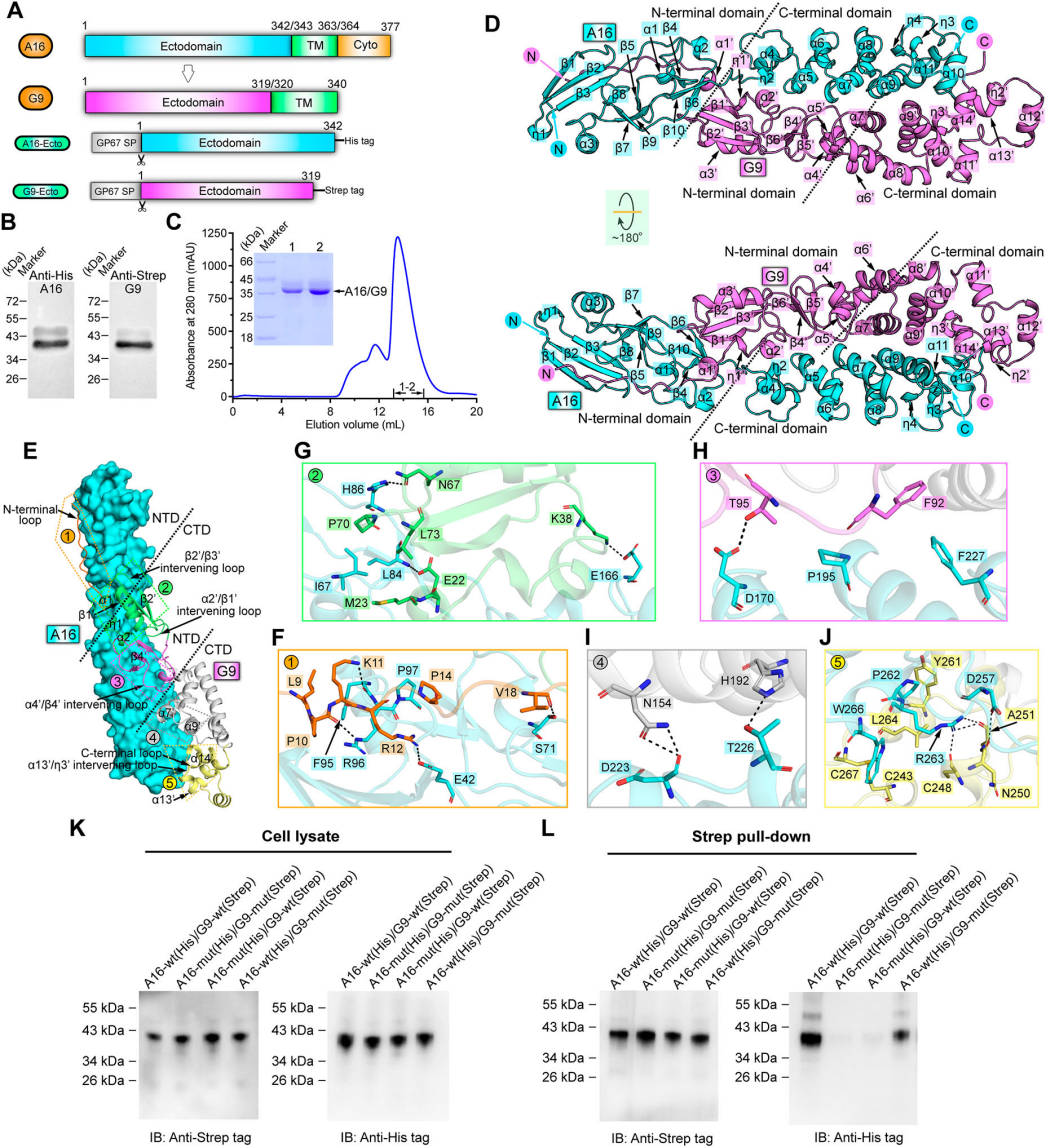
**Structure of vaccinia virus A16/G9 sub-complex.** (A) A schematic view of the protein-engineering strategy used to yield vaccinia virus A16-Ecto-His/G9-Ecto-Strep sub-complex. The transmembrane domain (TM), the ectodomain, and the cytoplasmic domain (Cyto) are individually marked with the boundary-residue numbers. (B) Identification of A16-Ecto-His/G9-Ecto-Strep hetero-complex using western blot assay. (C) Solution behaviour of A16-Ecto-His/G9-Ecto-Strep subcomplex on a Superdex 200 Increase 10/300 GL column. The inset figure shows the SDS-PAGE analysis of the pooled samples. (D) Overall structure of the hetero-dimer formed between A16 (cyan) and G9 (violet). The secondary structural elements are labelled. Both A16 and G9 could be subdivided into two domains, the boundary of which is highlighted with the dashed lines. (E-J) The atomic binding details between A16 and G9. The binding interface is subdivided into five patches (Patch1-Patch5) based on the G9 components involved in A16-engagement, which is depicted in panel (E). For clarity, only those residues providing important H-bond and hydrophobic interactions are shown and labelled. Dashed lines indicate hydrogen bonds. A full list of the inter-chain contacts, including H-bonds and vdw contacts, is summarized in Supplementary Table S2. (F) Amino acid interaction details in Patch1. (G) Amino acid interaction details in Patch2. (H) Amino acid interaction details in Patch3. (I) Amino acid interaction details in Patch4. (J) Amino acid interaction details in Patch5. (K-L) Verification of the A16/G9 interaction via mutagenesis. His-tagged A16 proteins (wt and mutant) and Strep-tagged G9 proteins (wt and mutant) were co-expressed in cells. G9 was then precipitated using the streptactin resin and the co-precipitated A16 was analysed and compared. (K) Western-blot analyses of the cell lysates. (L) Western-blot analyses of the pulled samples.

Within the crystallographic asymmetric unit of the structure, a single A16 protein and one G9 protein are present in a 1:1 binding mode. Traceable electron densities can be observed for A16 residues L7-C291 and G9 amino acids E8-N268 (Supplementary Figure S2). Both A16 and G9 feature with an extended rod-like fold. Their individual structures can be further subdivided into two domains: an α/β N-terminal domain (NTD) and a helical C-terminal domain (CTD) (Figure 1(D); Supplementary Figure S3).

For A16, its NTD is composed of ten β-strands (β1-β10), three α-helices (α1-α3) and one 3_10_-helix (η1). The strands assemble into three antiparallel β-sheets, which are further interspersed by the helical components to form a compact structure. The CTD of A16 consists of eight α-helices (α4-α11) and three 3_10_-helices (η2-η4). The helical elements of α5-α9 are arranged one-by-one in an anti-parallel mode, stacking into a two-layered helical array. This array is further decorated on one side by helices α4 and η2 and on the other by the intertwined helices α10-α11 and η3-η4, together assembling into a pure helical structure with an extended conformation. A bunch of intra-domain disulfide linkages are observed to form to further stabilize the individual domain structure, including two in NTD (C60/C90 and C70/C128) and seven in CTD (C146/C155, C147/C168, C176/C185, C204/C213, C236/C245, C247/C270 and C265/C291) (Figure 1(D); Supplementary Figure S3). Sterically, the NTD and CTD of A16 are linked via a relatively long loop, which might be of certain flexibility.

For G9, its NTD also features with an α/β structure. Within this domain, six β-strands (β1’-β6’) assemble into two anti-parallel β-sheets, which are further interspersed with four α-helices (α1’-α4’) and one additional 3_10_-helix (η1’). In addition, a long N-terminal loop is observed in the NTD of G9, extending out into A16-NTD. The CTD of G9 is composed of ten α-helices (α5’-α14’) and two 3_10_-helices (η2’-η3’). These helical components are intertwined together, once again assembling into a pure helical structure as observed for A16-CTD. The individual domain structure of G9 also features with intra-domain disulfides for stabilization, including one in NTD (C88/C117) and four in CTD (C135/C145, C177/C186, C223/C248 and C243/C268) (Figure 1(D); Supplementary Figure S3). Unlike that of A16, however, the inter-domain linker in G9 is of short length and an inter-domain disulfide-bond (C89/C127) is observed to form, dragging the two domains into close proximity (Supplementary Figure S3). We believe this would result in a limited domain-hinge plasticity between NTD and CTD of G9. It is notable that except for the angle of the inter-domain connections, the A16 and G9 structures predicted by AlphaFold2 seem to match the crystal structures well.

For the sub-complex formation, A16 and G9 both adopt a highly extended conformation. Resultantly, the two protein subunits are aligned almost in parallel, leading to good complementarity and the formation of an extended binding interface (Supplementary Figure S4). In order to characterize the atomic binding details between the two proteins, we further subdivided the binding interface into five patches (Patch1-Patch5) (Figure 1(E)) based on the G9 components involved in A16-engagement. Those residues located within the van der Waals (vdw) contact distance (4.5-Å-distance cutoff) between the two subunits were selected and listed in Supplementary Table S2. Patch1 mainly involves the N-terminal loop of G9-NTD. It extends over the surface of A16-NTD, positioning a linear array of G9-residues, including E8-R12 and P14-T20, for hydrogen-bond (H-bond), hydrophobic and vdw interactions with A16-residues L7, I10, I12, M24, E41-E42, F59, D68-H69, S71-F73, P76, F95-S99 and Y121 (Figure 1(F); Supplementary Table S2). Patch2 mainly involves the first β-sheet and its adjacent helices and loops in G9, which are positioned to the vicinity of the inter-domain loop in A16 (Figure 1(E)). Located within this patch include G9-residues D21-M23, L25, K29-V33, P36, K38, E41-Y42, H44, N67-G71 and L73, and A16-residues L61, I67-D68, S71, K80, V83-H86, Q126, K133, L136-S140, V143, I158-F159, T165-H167 and D170, providing H-bond, hydrophobic and vdw contacts (Figure 1(G); Supplementary Table S2). Patch3-Patch5 are all located in the CTD of A16. For G9, Patch3 involves its second β-sheet and the α4’/β4’ intervening loop in NTD, Patch4 mainly involves the α7’ and α9’ helices of CTD and Patch5 involves the α13’ and α14’ helices and several flanking loops in CTD (Figure 1(E)). Within these three patches, a large number of interface residues are identified for inter-molecule contacts, including G9-residues F92-N93 and T95-H96, and A16-residues D170, T173, R177, K194-P195, S198, D202 and F227 in Patch3 (Figure 1(H); Supplementary Table S2), G9-residues D120-H122, R142-N143, H146-Q147, G150-S151, N154, H191-H192, A195 and N197, and A16-residues E166, R193-K194, S198, S201, D223-F227, D229-T230, Y233-V234, Y282-N283 and R290 in Patch4 (Figure 1(I); Supplementary Table S2) and G9-residues C243, C248-A251, V253, Y261, L264-G265 and C267-N268, and A16-residues D257-K258, L260-R263, C265-W266, Q287 and C291 in Patch5 (Figure 1(J); Supplementary Table S2). It is noteworthy that the amino acids involved in vaccinia virus A16/G9 binding are highly conserved among members of the *Orthopoxvirus* genus, demonstrating that the orthopoxviruses, including the monkeypox virus, would share quite similar binding patterns and likely the same interaction details for A16/G9 sub-complex formation. It is also worth noting that the sub-complex of A16/G9, which is shown as a heterodimer and essentially folded to be α-helical, is distinct from currently-characterized three classes of viral fusogens. The well-known class I and class III viral fusion proteins are featured as homo-trimers, and the class II proteins are homo-/hetero-dimers but are largely of the β-structure [8].

We further performed mutagenesis study to validate the observed A16/G9 interaction. Because large number of amino acids and diverse types of inter-chain contacts (vdw contacts, hydrophobic interactions and H-bonds) are observed to locate along the A16-G9 interface, it is expected that single mutations or a small number of mutations in A16 and G9 proteins will not disrupt the tight binding of the two entities. Thus, a bunch of important residues involved in the interactions were simultaneously mutated into alanine. The subsequent A16 mutant (A16-mut) contains substitutions: E42A, I67A, S71A, L84A, H86A, F95A, R96A, E166A, D170A, T226A, D257A, P262A, R263A and W266A, and the G9 mutant (G9-mut) contains: L9A, P10A, K11A, R12A, E22A, M23A, K38A, N67A, P70A, T95A, N154A, H192A, Y261A and L264A. The A16 and G9 proteins were then co-expressed in pairs for co-precipitation analyses. Both the wild type and the mutant proteins were well expressed and extracted (Figure 1(K)). While A16-wt and G9-wt were readily co-purified, highly attenuated co-precipitation was observed for the A16-wt/G9-mut pair and no obvious co-precipitation was recorded for the A16-mut/G9-wt and A16-mut/G9-mut pairs (Figure 1(L)). The results demonstrated that those interface residues identified in our structure indeed play important roles in the A16/G9 sub-complex formation.

In conclusion, we report, to our knowledge, the first atomic structure of the poxvirus A16/G9 complex, which we believe would facilitate future studies on the mechanism of poxvirus EFC assembly and guide the antiviral drug design.

## Supplementary Material

Supplemental Material

## Data Availability

The datasets used and/or analysed during this study are available from the corresponding author on reasonable request. Atomic coordinates and structure factors for the reported crystal structure has been deposited into the Protein Data Bank under accession number 8GP6.
