# Supplementary material for: Structural basis of poxvirus A16/G9 binding for sub-complex formation
Source: Emerg Microbes Infect. 2023 Mar 1;12(1):2179351. doi: 10.1080/22221751.2023.2179351 (PMC9980159; doi:10.1080/22221751.2023.2179351)
Supplement: Supplemental Material [file TEMI_A_2179351_SM9014.docx]

Supplementary information for

**Structural basis of poxvirus A16/G9 binding for sub-complex formation**

Fanli Yang^1#^, Sheng Lin^1#^, Zimin Chen^1^, Dan Yue^1^, Ming Yang^1^, Bin He^1^, Yu Cao^1,2^, Haohao Dong^3^, Jian Li^4^, Qi Zhao^5^, Guangwen Lu^1*^

^1^ West China Hospital Emergency Department (WCHED), State Key Laboratory of Biotherapy, West China Hospital, Sichuan University, Chengdu, Sichuan 610041, China

^2^ Disaster Medicine Center, West China Hospital, Sichuan University, Chengdu, Sichuan 610041, China

^3^ Laboratory of Aging Research and Cancer Drug Target, State Key Laboratory of Biotherapy and Cancer Center, National Clinical Research Center for Geriatrics, West China Hospital, Sichuan University, Chengdu, Sichuan 610041, China

^4^ School of Basic Medical Sciences, Chengdu University, Chengdu, Sichuan 610106 China

^5^ College of Food and Biological Engineering, Chengdu University, Chengdu, Sichuan 610106 China

**# These authors contributed equally:** Fanli Yang, Sheng Lin

*** Correspondence:** Guangwen Lu ([lugw@scu.edu.cn](mailto:lugw@scu.edu.cn))

**Materials and Methods**

**Plasmid construction**

Vaccinia virus (Western Reserve strain) A16/G9 sub-complex was expressed using the Bac-to-Bac baculovirus expression system (Invitrogen). The coding sequences for the ectodomain of A16 (GenBank: AAO89415.1, amino acids M1-W342 with a C-terminal His-tag) and G9 (GenBank: AAO89366.1, amino acids M1-D319 with a C-terminal Strep-tag) were individually cloned into the pFastBac1 vector. For each construct, a previously described gp67 signal peptide sequence [1] was added to the protein N-terminus for protein secretion.

For the co-precipitation assay, the coding sequences of wild-type A16 ectodomain (with a C-terminal His-tag) and G9 ectodomain (with a C-terminal Strep-tag) were individually cloned into the pcDNA3.1 vector. The mutant plasmids were then constructed via mutagenesis. A16-mut contains mutations: E42A, I67A, S71A, L84A, H86A, F95A, R96A, E166A, D170A, T226A, D257A, P262A, R263A and W266A. G9-mut contains mutations: L9A, P10A, K11A, R12A, E22A, M23A, K38A, N67A, P70A, T95A, N154A, H192A, Y261A and L264A.

**Expression and purification of A16/G9 sub-complex**

Transfection and virus amplification of A16 and G9 were conducted with Sf9 cells respectively. For A16/G9 sub-complex expression, Hi5 cells were co-infected with the A16 and G9 baculoviral stocks simultaneously. The cell culture supernatants were collected at 96 hours after infection and passed through 5-ml HisTrap excel columns (GE Healthcare) for purification. The recovered protein preparations were further chromatographed onto Superdex 200 Increase 10/300 GL column (GE Healthcare) on protein purification system SDL-030-F2 (Sepure Instruments Inc.) with a buffer consisting of 20 mM Tris-HCl (pH 8.0) and 150 mM NaCl.

**Co-precipitation assay**

The 293T cells were pre-seeded in dishes for overnight culturing. When 70% confluency was obtained, the cells were co-transfected with the A16 (wild type or mutant) and G9 (wild type or mutant) expression plasmids in pairs. At 48 h post-transfection, the cells were lysed by cell lysis buffer (Beyotime Biotech. Inc.), supplemented by sonication. The debris of each sample was removed by centrifugation for 10 min at 12,000 rpm. Each lysate was incubated with 30 μl streptactin resin (GE Healthcare) on a rotator for 4 h. The supernatant was removed after 5-min centrifugation at 1,000 rpm. Then, the beads were washed four times with washing buffer [20 mM Tris-HCl (pH 8.0) and 500 mM NaCl]. Finally, the streptactin resin was re-suspended and directly subjected to western blot analyses.

**Western blot assay**

Firstly, the protein solutions were loaded and separated on an SDS-PAGE gel and transferred to polyvinylidene fluoride (PVDF) membranes. Then, the membranes were blocked with 5% non-fat milk in Tris-buffered saline containing 0.1% Tween-20 (TBS-T) for 2 h at room temperature, and incubated with primary antibodies against His-tag (1:2000) and Strep-tag (1:2000) overnight at 4°C, followed by incubation with horseradish peroxidase-conjugated secondary antibodies at 37°C for 1 h. Finally, the bands were detected using the enhanced chemiluminescence system.

**Crystallization**

Commercial crystallization kits (Molecular Dimensions and Hampton Research) were used for initial crystallization screening by the vapour-diffusion sitting-drop method. In brief, 1 µL A16/G9 protein solution was mixed with 1 µL reservoir solution, and the resultant mixture was then equilibrated against 90 µL reservoir solution at 18°C. Diffractable crystals were obtained under a condition consisting of 4% v/v (+/-)-2-Methyl-2,4-pentanediol, 0.1 M Citric acid pH (3.5) and 20% w/v PEG 1500.

**Data collection and structure determination**

For data collection, crystals were flash-cooled in liquid nitrogen after a brief soaking in reservoir solution supplemented with 20% (v/v) glycerol. Diffraction data were collected at Shanghai Synchrotron Radiation Facility (SSRF) beamline BL19U1 [2]. The collected data were then processed with HKL2000 [3] for indexing, integration and scaling. The sub-complex structure was solved by molecular replacement using PHASER [4] with the search templates (individual domains of A16 and G9 models) generated by AlphaFold2 [5]. Finally, the atomic models were completed with COOT [6] and refined with refmac5 [7] in CCP4. The final data processing and structure refinement statistics are summarized in Supplementary Table S1. All structural figures were generated using PyMOL (http://www.pymol.org).

**Sequences used in this study**

The GenBank accession numbers of the sequences (A16 and G9 homologues) used for structure-based multiple sequence alignment are as follows: VACV (vaccinia virus), GenBank: AAO89415.1 and AAO89366.1; MPXV (monkeypox virus), GenBank: URK20565.1 and URK20516.1; VARV (variola virus), GenBank: AAA60868.1 and AAA60820.1; CPXV (cowpox virus), GenBank: ADZ30329.1 and ADZ30280.1; CMLV (camelpox virus), GenBank: AAL73841.1 and AAL73792.1; AKMV (akhmeta virus), GenBank: AXN74931.1 and AXN74880.1; VPXV (volepox virus), GenBank: AOP31825.1 and AOP31776.1; ECTV (ectromelia virus), GenBank: AAM92424.1 and AAM92375.1; Abatino (abatino macacapox virus), GenBank: AYN64701.1 and AYN64653.1; RCNV (raccoonpox virus), GenBank: AKJ93765.1 and AKJ93716.1; SKPV (skunkpox virus), GenBank: AOP31614.1 and AOP31565.1; TATV (taterapox virus), GenBank: ABD97704.1 and ABD97656.1.

**References**

[1] Yang F, Lin S, Ye F, Yang J, Qi J, Chen Z, et al. Structural Analysis of Rabies Virus Glycoprotein Reveals pH-Dependent Conformational Changes and Interactions with a Neutralizing Antibody. Cell host & microbe. 2020; 27:1-13.

[2] Zhang W-Z, Tang J-C, Wang S-S, et al. The protein complex crystallography beamline (BL19U1) at the Shanghai Synchrotron Radiation Facility. *Nuclear Science and Techniques*. 2019;30(11):170.

[3] Otwinowski Z, Minor W. Processing of X-ray diffraction data collected in oscillation mode. *Methods Enzymol.* 1997;276:307-26.

[4] Read RJ. Pushing the boundaries of molecular replacement with maximum likelihood. *Acta Crystallogr D Biol Crystallogr*. 2001;57(Pt 10):1373-82.

[5] Zheng L, Meng J, Lin M, Lv R, Cheng H. et al*.* Structure prediction of the entire proteome of monkeypox variants. *Acta Mater Med*. 2022;1(2): 260-264. doi: 10.15212/AMM-2022-0017.

[6] Emsley P, Cowtan K. Coot: model-building tools for molecular graphics. *Acta Crystallogr D Biol Crystallogr*. 2004;60(Pt 12 Pt 1):2126-32.

[7] Kovalevskiy O, Nicholls RA, Long F, Carlon A, Murshudov GN. Overview of refinement procedures within REFMAC5: utilizing data from different sources. *Acta Crystallogr D Struct Biol*. 2018;74(Pt 3):215-27.


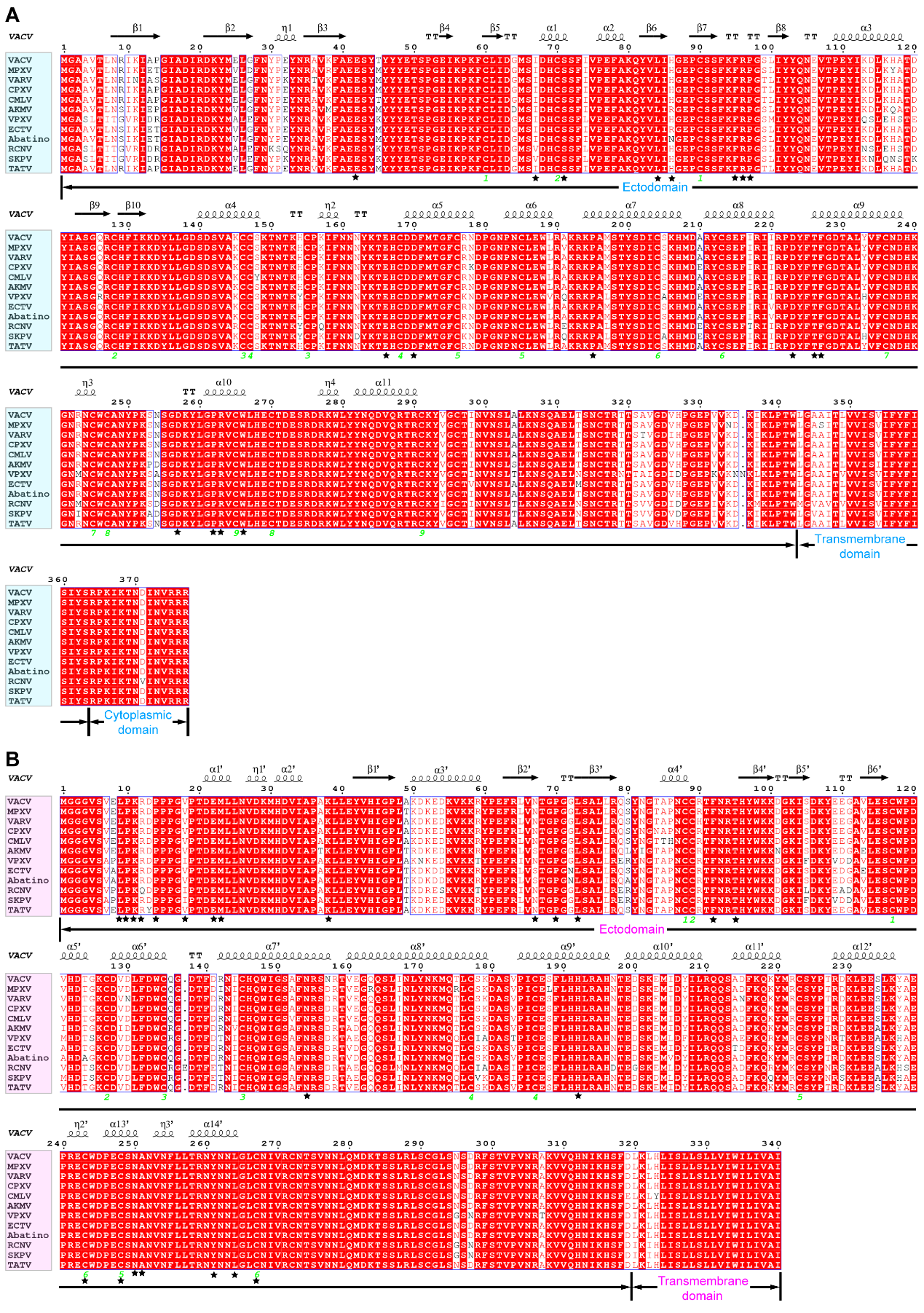


**Supplementary Figure S1.** Structure-based multiple sequence alignment of the A16 and G9 homologues from representative orthopoxviruses. (A) Multiple sequence alignment for A16 homologues. (B) Multiple sequence alignment for G9 homologues. The secondary structural elements are labelled above the sequences, with horizontal arrows indicating β-strands, spinal lines representing α-helices or 3_10_ helices and TT letters indicating turns. The transmembrane domain, the ectodomain, and the cytoplasmic domain are marked below the sequences. The disulfide-related cysteine residues are labelled with italic number in green, and disulfide bonds are formed between the two cysteines with the same number. Key residues involved in A16/G9 H-bond and hydrophobic interactions are highlighted with black stars. Abbreviations: VACV (vaccinia virus), MPXV (monkeypox virus), VARV (variola virus), CPXV (cowpox virus), CMLV (camelpox virus), AKMV (akhmeta virus), VPXV (volepox virus), ECTV (ectromelia virus), Abatino (abatino macacapox virus), RCNV (raccoonpox virus), SKPV (skunkpox virus), TATV (taterapox virus).


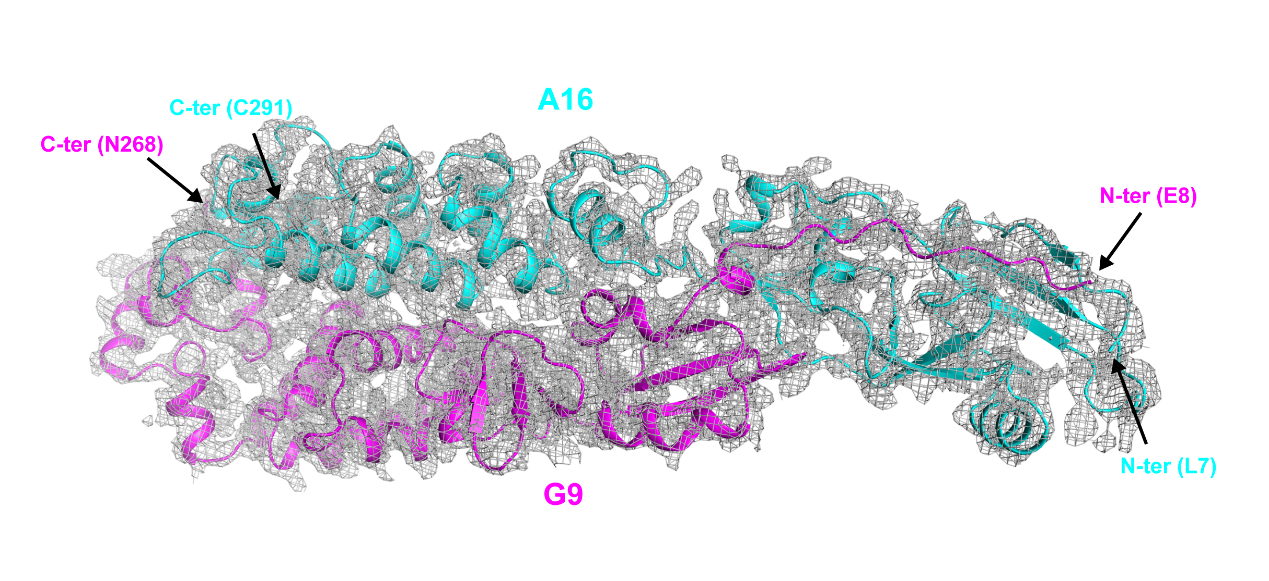


**Supplementary Figure S2.** Overview of the electron density map for the A16/G9 sub-complex structure. The densities are depicted by contouring using the 2 | Fo | - | Fc | map at 1.0 σ. The terminal residues are labelled.

**
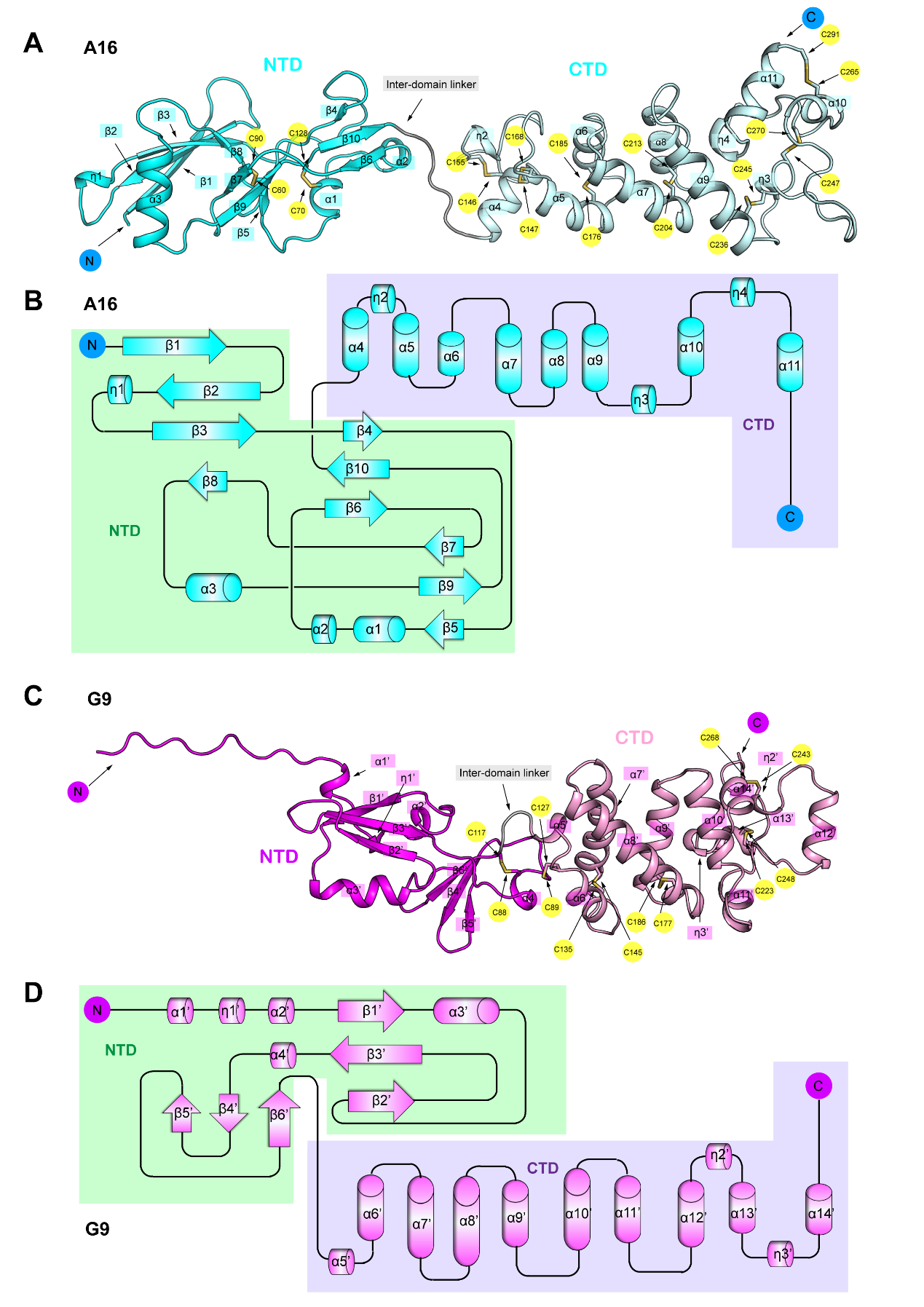
**

**Supplementary Figure S3.** Overview of the organization of secondary structural elements and the topological diagrams of A16 and G9. Both A16 and G9 could be subdivided into two domains: an α/β N-terminal domain (NTD) and a helical C-terminal domain (CTD). For clarity, A16-NTD, A16-CTD, G9-NTD and G9-CTD are highlighted in cyan, pale cyan, magenta and light pink, respectively. The secondary structural elements and the disulfide linkages are labelled. Letter C in yellow circle refers to cysteine residue. (A) Structural presentation of A16 for detailed organization of secondary structural elements. (B) A topological diagram of A16. (C) Structural presentation of G9 for detailed organization of secondary structural elements. (D) A topological diagram of G9.

**
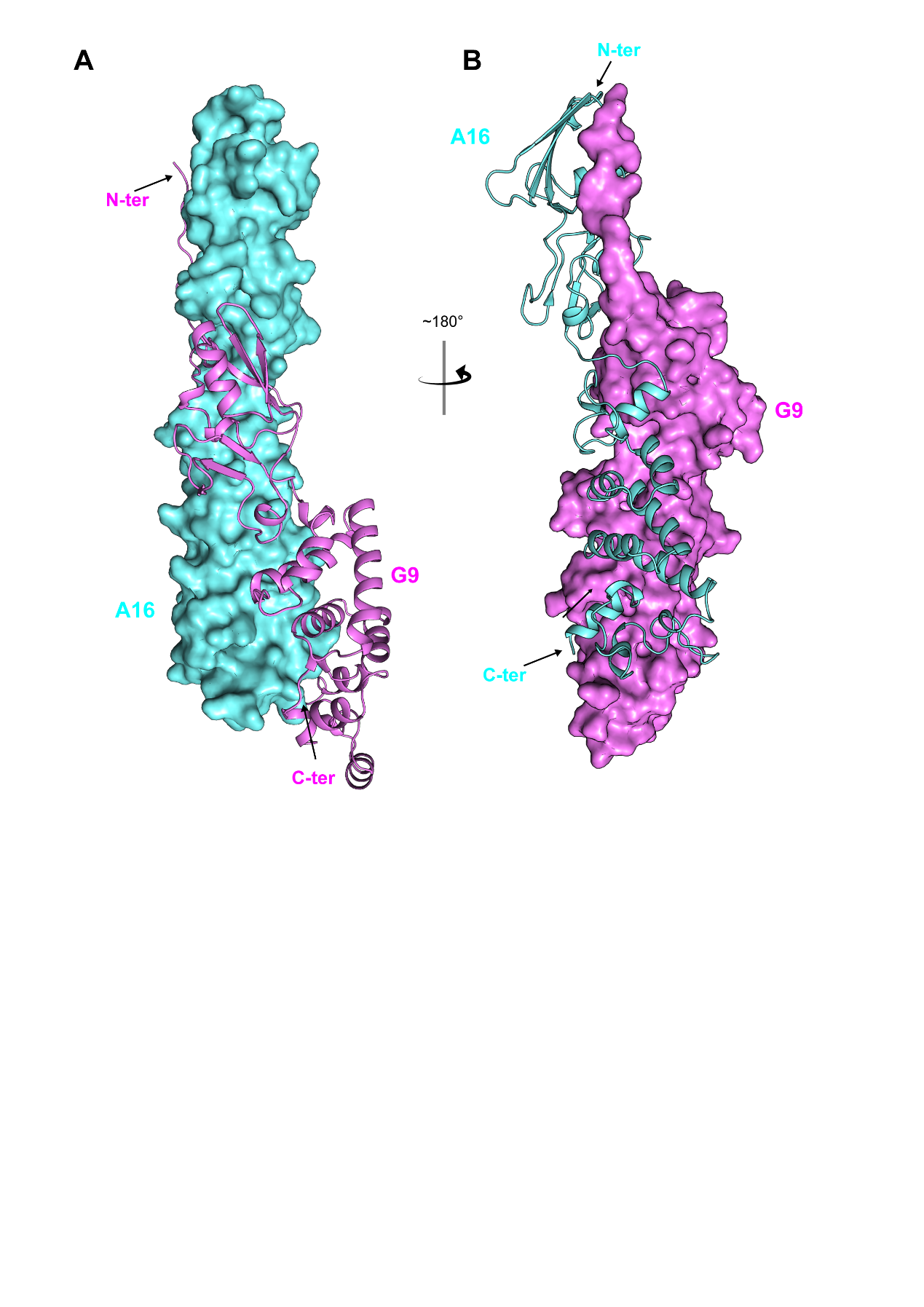
**

**Supplementary Figure S4.** Shape complementarity between A16 and G9 upon complex formation. (A) A16 and G9 are shown in surface and cartoon representations, respectively. (B) A16 and G9 are shown in cartoon and surface representations, respectively.

**Supplementary Table S1.** Data collection and structure refinement statistics.

|  | Vaccinia virus A16/G9 |
| --- | --- |
| **Data collection** |  |
| Space group | *P*22_1_2_1_ |
| Cell dimensions |  |
| *a*, *b*, *c* (Å) | 41.28, 122.01, 123.91 |
| *α*, *β*, *γ* (°)  Wavelength (Å) | 90, 90, 90  0.97890 |
| Resolution (Å) | 50.00-2.70 (2.80-2.70) |
| Unique reﬂections | 18033 (1775) |
| *R*_merge_ | 0.096 (0.613) |
| *I*/sig*I* | 26.70 (3.58) |
| Completeness (%) | 99.9 (99.8) |
| Redundancy | 12.3 (12.3) |
|  |  |
| **Refinement** |  |
| Resolution (Å) | 30.00-2.70 |
| No. reflections | 17099 |
| *R*_work_/*R*_free_ | 0.243/0.273 |
| No. of atoms |  |
| Protein | 4456 |
| Ligand/ion | 6 |
| Water | 0 |
| *B*-factors (Å^2^) |  |
| Protein | 68.58 |
| Ligand/ion | 69.75 |
| Water | - |
| R.m.s. deviations |  |
| Bond lengths (Å) | 0.0118 |
| Bond angles (°) | 1.5838 |
| Ramachandran plot (%)  Favored region  Allowed region  Outlier region | 95.6  4.4  0 |
| **PDB code** | 8GP6 |

A single crystal was used to collect the data.

Values in parentheses are for the highest-resolution shell.

**Supplementary Table S2.** Detailed atomic interaction between vaccinia virus A16 and G9.

| G9^a^ | | Contacts with A16 amino acids^b,c^ | Total contacts^d^ |
| --- | --- | --- | --- |
| Patch1 | E8 | L7 (1), I10 (3) | 218 |
|  | L9 | I10 (3), F95 (7), Y121 (1) |  |
|  | P10 | I10 (4), I12 (1), M24 (2), F95 (6), R96 (9) [1] |  |
|  | K11 | F95 (14) [1], R96 (3) |  |
|  | R12 | E41 (15), E42 (6) [1], F95 (2), R96 (28), P97 (4), G98 (2), S99 (1) |  |
|  | P14 | F59 (2), H69 (2), P97 (9) |  |
|  | P15 | E42 (4), F59 (1), H69 (4), S72 (3), F73 (5) |  |
|  | P16 | H69 (1), S72 (4) |  |
|  | G17 | D68 (12), H69 (8), S72 (2) |  |
|  | V18 | D68 (11), S71 (9) [1], S72 (11), P76 (1) |  |
|  | P19 | D68 (8), S71 (6) |  |
|  | T20 | S71 (3) |  |
| Patch2 | D21 | K80 (9) | 250 |
|  | E22 | V83 (10), L84 (6) [1], L136 (7) |  |
|  | M23 | I67 (9), D68 (8), S71 (1) |  |
|  | L25 | L136 (4), G138 (4) |  |
|  | K29 | G138 (4), D139 (5), S140 (2) |  |
|  | M30 | L137 (1), G138 (5) |  |
|  | H31 | S140 (2) |  |
|  | D32 | S140 (9), V143 (4), F159 (1) |  |
|  | V33 | L136 (1), L137 (5), G138 (2), D139 (3), V143 (3), F159 (2) |  |
|  | P36 | F159 (2), H167 (9), D170 (5) |  |
|  | K38 | T165 (2), E166 (7) [1], H167 (5) |  |
|  | E41 | I158 (2), H167 (6) |  |
|  | Y42 | K133 (3) |  |
|  | H44 | V83 (10), K133 (4), L136 (3) |  |
|  | N67 | H86 (6) [1] |  |
|  | T68 | L84 (3), I85 (5), H86 (5) |  |
|  | G69 | L84 (2), I85 (2), H86 (16) |  |
|  | P70 | L61 (2), I67 (7), L84 (5), I85 (3), H86 (9), Q126 (8) |  |
|  | G71 | I67 (5) |  |
|  | L73 | I67 (1), L84 (6) |  |
| Patch3 | F92 | K194 (9), P195 (6), S198 (9), F227 (5) | 56 |
|  | N93 | T173 (1), R177 (5), P195 (2), S198 (3), D202 (2) |  |
|  | T95 | D170 (4) [1], P195 (1) |  |
|  | H96 | D170 (9) |  |
| Patch4 | D120 | E166 (1), R193 (3), K194 (5) | 157 |
|  | V121 | K194 (1) |  |
|  | H122 | K194 (3) |  |
|  | R142 | Y233 (28) |  |
|  | N143 | T230 (15), V234 (3) |  |
|  | H146 | T226 (4), T230 (1) |  |
|  | Q147 | S198 (1), S201 (1), T226 (3), F227 (13), T230 (6) |  |
|  | G150 | T226 (5) |  |
|  | S151 | F227 (2) |  |
|  | N154 | D223 (9) [2], Y224 (22) |  |
|  | H191 | D229 (1), N283 (9) |  |
|  | H192 | F225 (5), T226 (5) [1] |  |
|  | A195 | F225 (3), Y282 (1) |  |
|  | N197 | R290 (7) |  |
| Patch5 | C243 | W266 (9) | 113 |
|  | C248 | R263 (4) [1] |  |
|  | S249 | K258 (1), R263 (11) |  |
|  | N250 | D257 (3) [1], R263 (7) |  |
|  | A251 | D257 (2), R263 (9) [2] |  |
|  | V253 | L260 (1), P262 (2), R263 (3) |  |
|  | Y261 | G261 (1), P262 (17), Q287 (5) |  |
|  | L264 | P262 (9), R263 (1), W266 (2) |  |
|  | G265 | P262 (4), C291 (1) |  |
|  | C267 | W266 (12) |  |
|  | N268 | C265 (1), W266 (8) |  |

^a^The interface residues in G9 and the ‘binding patch’ to which these amino acids belong are listed (the distance cutoff is 4.5 Å).

^b^Numbers in parentheses represent the number of van der Waals contacts between the indicated G9 and A16 residues (the distance cutoff is 4.5 Å).

^c^Numbers in brackets represent the number of hydrogen bonds between the indicated G9 and A16 residues (the distance cutoff is 3.2 Å).

^d^Total contacts indicate the sum of the van der Waals contacts for each patch listed in the first column.
